# Cysteine Catabolism: A Novel Metabolic Pathway Contributing to Glioblastoma Growth

**DOI:** 10.1158/0008-5472.CAN-13-1423

**Published:** 2013-12-18

**Authors:** Antony Prabhu, Bhaswati Sarcar, Soumen Kahali, Zhigang Yuan, Joseph J. Johnson, Klaus-Peter Adam, Elizabeth Kensicki, Prakash Chinnaiyan

**Affiliations:** 1 Radiation Oncology, H. Lee Moffitt Cancer Center and Research Institute, Tampa, Florida; 2 Chemical Biology and Molecular Medicine, H. Lee Moffitt Cancer Center and Research Institute, Tampa, Florida; 3 Advanced Microscopy Laboratory, H. Lee Moffitt Cancer Center and Research Institute, Tampa, Florida; 4 Cancer Imaging and Metabolism, H. Lee Moffitt Cancer Center and Research Institute, Tampa, Florida; 5 Metabolon, Inc., Durham, North Carolina

## Abstract

The relevance of cysteine metabolism in cancer has gained considerable interest in recent years, largely focusing on its role in generating the antioxidant glutathione. Through metabolomic profiling using a combination of high-throughput liquid and gas chromatography–based mass spectrometry on a total of 69 patient-derived glioma specimens, this report documents the discovery of a parallel pathway involving cysteine catabolism that results in the accumulation of cysteine sulfinic acid (CSA) in glioblastoma. These studies identified CSA to rank as one of the top metabolites differentiating glioblastoma from low-grade glioma. There was strong intratumoral concordance of CSA levels with expression of its biosynthetic enzyme cysteine dioxygenase 1 (CDO1). Studies designed to determine the biologic consequence of this metabolic pathway identified its capacity to inhibit oxidative phosphorylation in glioblastoma cells, which was determined by decreased cellular respiration, decreased ATP production, and increased mitochondrial membrane potential following pathway activation. CSA-induced attenuation of oxidative phosphorylation was attributed to inhibition of the regulatory enzyme pyruvate dehydrogenase. Studies performed *in vivo* abrogating the CDO1/CSA axis using a lentiviral-mediated short hairpin RNA approach resulted in significant tumor growth inhibition in a glioblastoma mouse model, supporting the potential for this metabolic pathway to serve as a therapeutic target. Collectively, we identified a novel, targetable metabolic pathway involving cysteine catabolism contributing to the growth of aggressive high-grade gliomas. These findings serve as a framework for future investigations designed to more comprehensively determine the clinical application of this metabolic pathway and its contributory role in tumorigenesis.

## Introduction

Gliomas represent a common type of primary brain tumor that arises from glial cells in the brain, which includes astrocytes, oligodendrocytes, and ependymal cells. The World Health Organization classifies glioma into grades 1 to 4 based on specific pathologic criteria, which play a central role in prognosis and clinical management. For example, patients with grade 1 gliomas are typically cured following surgical resection, whereas patients diagnosed with grade 4 gliomas, also termed glioblastoma, have a median survival of approximately 1 year despite aggressive multimodality therapy (1, 2). Considerable progress has been made in understanding the underlying biology of gliomas. For example, common molecular alterations identified in low-grade oligodendrogliomas and astrocytomas are allelic loss of 1p and 19q and mutations in p53, respectively, whereas grade 3 and 4 astrocytomas typically are driven by alterations in phosphoinositide 3-kinase (PI3K), EGFR, VEGF, and PTEN signaling (2). More recently, mutations in the metabolic enzyme isocitrate dehydrogenase 1 (IDH1) have been identified in low-grade glioma and secondary glioblastoma, which were then discovered to form the oncometabolite 2-hydroxyglutarate (2-HG) that demonstrated the capacity to regulate global epigenetic programs in these tumors (3–5).

Despite these advancements in understanding glioma biology, the underlying metabolic alterations that drive the aggressive phenotype of glioblastoma remain unclear. Nearly a century ago, Warburg and colleagues made the seminal observation of aerobic glycolysis in cancer (6, 7), yet a definitive explanation for why tumor cells metabolize glucose through this seemingly inefficient process and the selective advantage offered remains unclear. However, its clear relevance is evident with the widespread application of 18-FDG–PET (2[18F]fluoro-2-deoxy-D-glucose–positron emission tomography) imaging, which can also predict histologic grade in glioma with relatively high accuracy (8).

A comprehensive study of glioma metabolism was recently performed using global metabolomic profiling on patient-derived tumors. These investigations identified unique metabolic subtypes in glioma, with the metabolic signature of glioblastoma being consistent with anabolic metabolism (9). Through this line of investigation, we identified the metabolic intermediate of cystine catabolism cysteine sulfinic acid (CSA) as a novel metabolite associated with glioblastoma, demonstrating a more than 23-fold increase in accumulation when compared with grade 2 glioma. Cysteine is a semiessential amino acid that plays an important role in the metabolic cross-talk between neurons and astrocytes. It is subsequently metabolized, resulting in the synthesis of either taurine or glutathione, which have established roles as essential intermediates for brain function (10, 11). Although the accumulation of taurine has been demonstrated in astroglial cells, this metabolite does not seem to be relevant in glioma (10, 12). Rather, our current understanding of cysteine metabolism in relation to tumor biology has primarily focused on its role in glutathione (GSH) synthesis. Specifically, cysteine serves as the rate-limiting intermediary in the formation of GSH, which is one of the most abundant antioxidants in the central nervous system (13, 14). GSH synthesis through cystine uptake has been previously described to play an important role in glioma cell survival during redox stress and hypoxia and modulating this metabolic pathway has demonstrated therapeutic potential (13). Although the generation of the antioxidant GSH from cysteine, and its contributory role in modulating redox status, has been studied in detail, this alternate catabolic pathway leading to the accumulation of CSA has yet to be identified in tumors. In this report, we present for the first time the identification and potential relevance this metabolic pathway may play in glioblastoma biology.

## Materials and Methods

### Tumor samples and patient characteristics

Tumor specimens used in these studies are as described previously (9). Briefly, all surgeries were performed at the H. Lee Moffitt Cancer Center and Research Institute (Tampa, FL) and tissue was obtained from the Moffitt Cancer Center Tissue Core Facility. All of the grade 3 and 4 tumors used in this analysis were newly diagnosed malignancies. Tumors were fresh-frozen and their integrity and histology confirmed by a staff pathologist before aliquoting samples. Institutional Review Board/Human Subjects approval was obtained for this retrospective study (MCC16197).

### Global metabolomics and targeted metabolite quantitation

Metabolomic studies were conducted at Metabolon Inc. using methods previously described (9). Briefly, metabolic profiling analysis combined three independent platforms: ultrahigh performance liquid chromatography/tandem mass spectrometry (UHPLC/MS-MS) optimized for basic species, UHPLC/MS-MS optimized for acidic species, and gas chromatography/mass spectrometry (GC/MS). Metabolites were identified by automated comparison of the ion features in the experimental samples to a reference library of chemical standard entries that included retention time, molecular weight (*m/z*), preferred adducts, and in-source fragments as well as associated MS spectra, and were curated by visual inspection for quality control using software developed at Metabolon (15). Statistical analysis of log-transformed data was performed using “*R*” (16) and Welch *t* tests were performed to compare data between experimental groups. Multiple comparisons were accounted for by estimating the false discovery rate using *q* values (17).

Absolute quantitation of CSA was analyzed by isotope dilution UHPLC/MS-MS. Calibration and internal standard solutions were prepared in water. Tissue (30 ± 10 mg) was spiked with 50 μL internal standard solution (10 μg/mL CSA-D_4_, prepared according to Santhosh-Kumar and colleagues (18), 70 μL of water and 300 μL of acetonitrile and simultaneously homogenized/extracted in the presence of beads in a Geno-Grinder 2010. Following centrifugation, 5 μL of the supernatant was analyzed on a Waters Acquity/Thermo Quantum Ultra LC/MS-MS equipped with a Ascentis Express HPLC HILIC, 2.7 μm, 2.1 × 50 mm column (Supelco) using a 0.5% formic acid in water/acetonitrile gradient. Mass spectrometer conditions were selective reaction monitoring, negative ionization mode, and HESI source, and monitored transitions were CSA, *m/z* 152 ≥ 88; CSA-D_4_, *m/z* 156 ≥ 92. Quantitation was performed using a weighted linear least squares regression analysis generated from 10 calibration standards (0.01 to 10 μg/mL).

### Cysteine dioxygenase 1 expression

Cysteine dioxygenase 1 (CDO1) expression was evaluated by Western blot analysis and immunohistochemistry (IHC). Western blot assays were performed using antibodies against CDO1 (Abcam; ab53436). Blots were quantified using ImageJ (NIH, Bethesda, MD), and the CDO1 expression of individual samples was normalized to loading control (β-actin). Immunohistochemical staining was used to determine CDO1 expression on a glioma tissue microarray purchased from US Biomax (GL 103a). The slides were stained using the Ventana Discovery XT automated system (Ventana Medical Systems) following the manufacturer's protocol and counter stained with hematoxylin. Slides were scanned using the Aperio ScanScope XT and Image analysis to quantify CDO1 expression was performed using a Aperio Nuclear v9.1 algorithm as previously described (19).

### Cell culture

Human glioblastoma cell lines U251, T98G, and U87 were obtained from American Type Culture Collection (ATCC) and grown in RPMI-1640 (U251; GIBCO) or Eagle Minimum Essential Medium (U87 and T98G; ATCC) supplemented with 10% inactivated FBS. The glioblastoma neural stem cell line G179 was provided by Dr. Austin Smith (University of Cambridge, Cambridge, United Kingdom), distributed by BioRep (20), and grown in conditions as previously described (19). Cell line authentication was not carried out by authors within the last 6 months.

### Establishment of stable CDO1 knockdown cells

U251 cell clones using short hairpin RNA (shRNA) lentiviral particles (sc-91642-v; Santa Cruz Biotechnology). The shRNA–control group was transfected by control shRNA lentiviral particles (LP-Neg-LV 105-0200; Genecopoeia Inc.). Cells were seeded in 12-well plates, grown to 80% confluency, and infected with shRNA more than 48 hours in the presence of polybrene (5 μg/mL; Santa Cruz Biotechnology) according to the manufacturer's protocol. Stable colonies were selected and isolated in the presence of puromycin (2 μg/mL; Sigma).

### Oxygen consumption rate

Real-time oxygen consumption rate (OCR) was measured using the Seahorse Extracellular Flux Analyzer (XF24; Seahorse Bioscience). Cells were seeded in their respective media overnight. One hour before the experiment, cells were washed and media replaced with Seahorse assay media. Indicated compounds or vehicle controls were injected directly to the wells at the stated concentrations through pneumatic injection ports. For sulfasalazine experiments, cells were pretreated (200 μmol/L) 6 hours before each experiment. Readings were normalized to total protein and data analyzed using Seahorse software, with the unpaired *t* test being applied to test the significance of change.

### Mitochondrial isolation

Approximately 150 × 10^6^ cells were used to isolate mitochondria. Cells were gently scraped in ice-cold PBS and spun at 600 × *g* at 4°C for 10 minutes. The supernatant was discarded and the cells were resuspended in 3 mL of ice-cold isolation buffer (10 mL of 0.1 mol/L Tris–MOPS, 1 mL of EGTA/Tris, 20 mL of 1 mol/L sucrose in 69 mL of distilled water, pH 7.4). Cells were then homogenized using a precooled Teflon pestle in a glass potter. The homogenate was then spun at 600 × *g* at 4°C for 10 minutes. The supernatant was transferred to a glass centrifuge tube and spun at 7,000 × *g* at 4°C for 10 minutes. The supernatant was discarded and the mitochondrial pellet was resuspended in ice-cold isolation buffer. The mitochondrial concentration was measured by the Biuret method and the concentration was adjusted approximately to 50 mg/mL.

### Mitochondrial membrane potential

The JC-1 Assay Kit (Cayman Chemical Company) was used according to the manufacturer's protocol. Micrographs of images were taken with a Leica TCS SP5 AOBS laser scanning confocal microscope through a 63X/1.4NA Plan Apochromat oil immersion objective lens (Leica Microsystems CMS GmbH). Of note, 405 Diode, Argon 488, and HeNe 543 laser lines were applied to excite the samples and tunable emissions were used to minimize cross-talk between fluorochromes. Images for each sample were captured with photomultiplier detectors and prepared with the LAS AF software version 2.6 (Leica Microsystems).

### Pyruvate dehydrogenase activity

A pyruvate dehydrogenase (PDH) enzyme activity microplate assay kit (Abcam; ab109902) was used. Cells were grown to 80% confluency in 100-mm dish. After treatments the cells were placed into ice-cold PBS, and the manufacturer's protocol was followed.

### ATP quantification

The ATP determination Kit (Invitrogen) was used. Cells were grown to 80% confluency in 100-mm dish. After the respective treatments, cells were placed into ice-cold PBS and the manufacturer's protocol was followed.

### Apoptosis

A caspase-3 apoptosis kit (BD Biosciences) was used and the manufacturer's protocol was followed and analyzed by fluorescence-activated cell sorting (FACS).

### Redox stress

An H_2_-DCFDA (dihdyrodichlorofluorescein, diacetate) kit from Invitrogen was used and analyzed by FACS according to the manufacturer's protocol.

### Animal handling

All *in vivo* experiments were performed according to the institutional guidelines and approved by the Institutional Animal Care and Use Committee (4031R). Xenograft tumors were established in athymic nu/nu mice (Charles River Laboratories) using methods previously described (19). For intracranial tumors, mice were anesthetized, indicated cell suspensions (10^6^ cells in 10 μL) were injected using a stereotactic device, and sacrificed when neurologic symptoms became apparent. Kaplan–Meier curves were used and a log-rank value was calculated to analyze survival.

### Statistical analysis

Statistical analysis was done using a Student *t* test unless otherwise indicated.

## Results

### Tumor grade–specific changes of CSA levels and CDO1 expression in glioma

**Figure 1:**
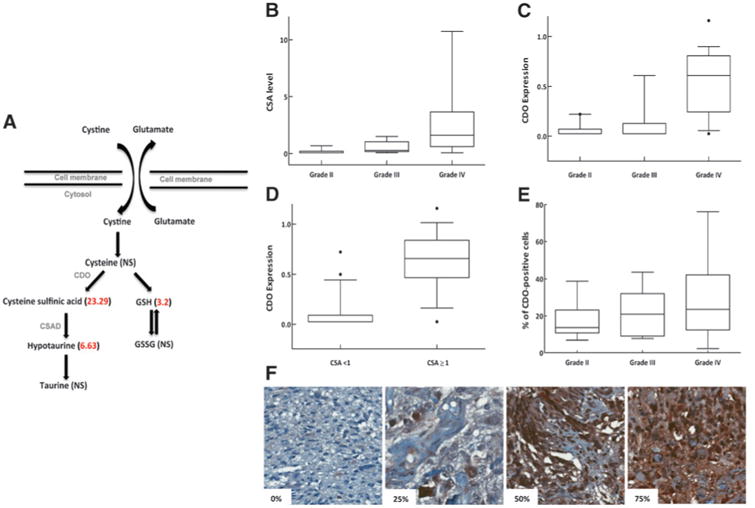
Cysteine metabolism in glioma. A, schematic of cysteine metabolism in glioma. Numbers in parenthesis reflect fold-change increases when comparing metabolite levels between grade 4 to grade 2 gliomas. B, grade-specific CSA levels in glioma. Grade 2 versus 3, *P* = 0.001; grade 3 versus 4, *P* < 0.001; grade 2 versus 4, *P* < 0.001. C, grade-specific changes of CDO1 expression in glioma determined by Western blot analysis and normalized to β-actin. Grade 2/3 versus 4; *P* < 0.001. D, intrasample concordance of CSA levels and CDO expression (*P* < 0.001). E, grade-specific changes of CDO expression in glioma determined by IHC. Grade 2 versus 4, *P* = 0.06. F, representative pictographs of CDO positivity in glioma. CSAD, CSA dioxygenase; GSSG, oxidized glutathiones. *, outliers.

Global metabolomic profiling was performed using a combination of UHPLC/MS-MS and GC/MS on a total of 69 fresh-frozen glioma specimens surgically resected (18 grade 2, 18 grade 3, and 33 grade 4) using a metabolomic library consisting of more than 2,000 purified standards. Following log transformation and imputation with minimum observed values for each compound, Welch two-sample *t* tests were used to identify biochemicals that differed significantly between histologic grades. These studies identified metabolites associated with cysteine metabolism as a significant metabolic pathway–differentiating grade 2 and grade 4 glioma (Fig. 1A). Cystine uptake in glioma cells is primarily attributed to the membrane transporter system x_c_^−^, which mediates the exchange of extracellular cystine with intracellular glutamate (21). Cystine is then rapidly reduced intracellularly to cysteine, in which it forms GSH when combined with glutamate and glycine, or is further metabolized to taurine through the metabolic intermediaries CSA and hypotaurine. Both GSH and hypotaurine demonstrated significant differences between glioma grade, which were 3.2- (*P* = 0.01) and 6.63-fold (*P* < 0.001) higher in glioblastoma, respectively. However, these studies identified a more than 23-fold increase in CSA levels in glioblastoma when compared with grade 2 glioma (*P* < 0.001), which ranked as the metabolite with the highest relative accumulation in glioblastoma when compared with grade 2 glioma (Fig. 1A and B). Supplementary Table S1 provides a list of the top 10 metabolites with highest fold increases and decreases in glioblastoma when compared with grade 2 glioma. We went on to both validate and quantify CSA levels in glioblastoma using targeted UHPLC/MS-MS in a panel of tumors originally profiled that demonstrated a range of CSA levels, from modest (∼2 fold) to high (∼10 fold) increases in CSA accumulation. Quantification in samples demonstrating 1.95- to 10.32-fold increases in CSA levels corresponded to CSA concentrations ranging from 2.35 to 25.8 ng/mg in tissue with a relatively high intrasample concordance (Supplementary Fig. S1; *r*^2^ = 0.84).

As cysteine catabolism along this metabolic pathway has not been previously studied in the context of tumor biology, as an initial investigation, we tested the hypothesis that CDO1, which metabolizes CSA from cysteine, would be aberrantly expressed in high-grade glioma. Of the initial 69 tumors profiled, 34 had tissue available for further analysis. When normalized to β-actin, Western blot analysis performed on tissue lysates demonstrated a significant increase in CDO1 expression in grade 4 glioma (Fig. 1C; Western blots provided in Supplementary Fig. S2). Tumors with CSA levels ≥1 (representing the median value) demonstrated a significant increase in CDO1 expression (Fig. 1D), suggesting intratumoral concordance between CSA levels and CDO1 expression and further supports cysteine catabolism along this parallel pathway is a relevant event in glioblastoma. CDO1 expression was then determined in an independent dataset using immunohistochemical staining on a glioma tumor microarray. Automated image analysis software was used to determine the number of positive cells in each specimen. Grade 4 glioma demonstrated a trend in higher positivity when compared with grade 2 and 3 (*P* = 0.06). Although intragrade heterogeneity of CDO1 expression was identified in glioblastoma, with a quartile of tumors demonstrating low CDO1 expression, high expression of CDO1 (>40%) was observed exclusively in grade 4 tumors, a pattern that was present in a quartile of tumors (Fig. 1E). Representative pictographs illustrating 0% to 75% staining of CDO1 are provided in Fig. 1F.

### CSA modulates mitochondrial function

**Figure 2:**
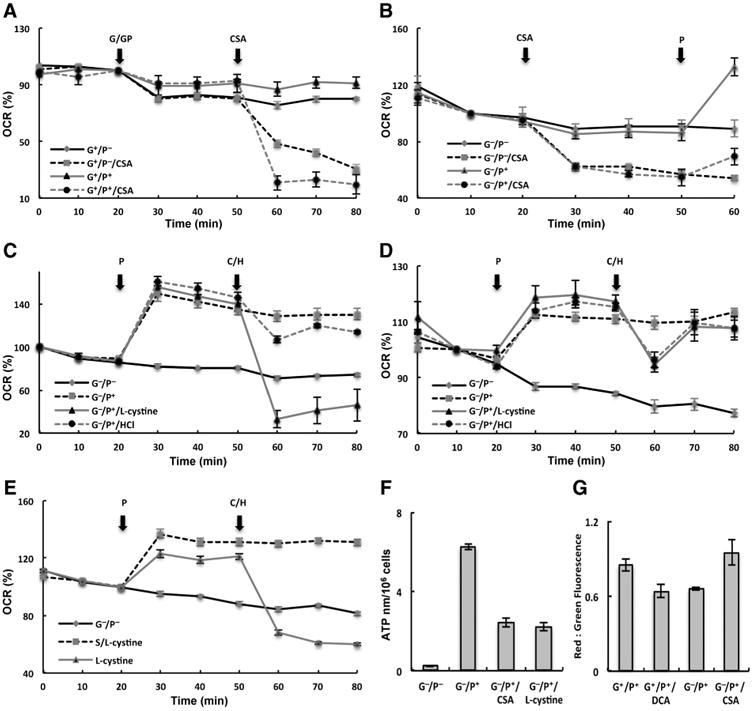
CSA modulates mitochondrial function in glioblastoma cells. A, CSA (1 mmol/L) attenuates OCR in G179 cells in described conditions. G^+^/P^−^ (*P* = 0.003); G^+^/P^+^ (*P* < 0.001). B, pretreatment of CSA attenuates pyruvate-induced OCR (*P* = 0.001). C, l-cystine attenuates OCR in U251 shControl cells (*P* = 0.008); however, this was abrogated (D) when evaluated in CDO1 stable knockdown cells (shCDO; *P* = 0.72). E, pretreatment of cells with the system xc^−^ inhibitor sulfasalazine (200 mmol/L) attenuates l-cystine-induced decrease in OCR (*P* < 0.001). F and G, CSA(1 mmol/L) and l-cystine (100 μmol/L) attenuates ATP synthesis (*P* < 0.001; F) and increases mitochondrial potential (determined by an increase in red:green ratio in the JC-1 assay; G) in U251 (*P* = 0.012). Results are representative of at least three independent experiments. G, glucose; P, pyruvate; C, l-cystine; H, HCl; S, sulfasalazine.

As the role of CSA in gliomagenesis has not yet been explored, our initial studies focused on general cellular metabolism using the Seahorse XF Extracellular Flux Analyzer. Exogenous exposure of CSA consistently resulted in decreased cellular oxygen consumption in varying treatment conditions in the glioblastoma neural stem cell line G179 (Fig. 2A and Supplementary Fig. S3A; ref. 20). This observation was particularly notable in treatment conditions consisting of pyruvate addition to promote aerobic respiration. Similar findings were observed when we extended our studies to established glioblastoma cell lines U251 and T98G (Supplementary Fig. S3B). On the basis of the initial observation of CSA attenuating cellular respiration, we sought to determine whether pretreating cells with CSA could modulate pyruvate-induced oxygen consumption. As demonstrated in Fig. 2B, CSA exposed to cells in glucose- and pyruvate-deficient conditions decreased mitochondrial oxygen consumption by 36% ± 1%. As expected, the addition of pyruvate increased oxygen consumption (54% ± 3%); however, this increase was attenuated in cells exposed to CSA only demonstrating a 25% ± 3% increase in oxygen consumption (*P* < 0.001), further supporting CSA's role in modulating mitochondrial respiration. Next, we attempted to recapitulate the observed changes in cellular respiration through activation of the metabolic pathway involved in CSA biosynthesis. We first evaluated CDO1 expression, the enzyme involved in CSA synthesis, in a panel of glioblastoma lines. Consistent with tissue samples, CDO1 was differentially expressed in cell lines, with relatively high levels of expression in U251 and G179 cells, and little to no expression in T98G and U87 (Supplementary Fig. S4A). We then sought to determine whether the upstream metabolite responsible for CSA biosynthesis, l-cystine, which has a well-established role in cross-talk between astrocytes and neurons, could be metabolized through this pathway to have a similar effect on cellular respiration as CSA Because l-cystine requires a pH of 2.0 to be soluble, a pH control was used in these studies. As demonstrated in Fig. 2C, l-cystine (100 μmol/L) had a similar effect of attenuating cellular respiration as CSA in the CDO1-expressing cell line U251. To more definitively attribute the metabolic pathway associated with CSA accumulation to the observed changes in cellular respiration, we generated a stable knockdown of CDO1 in U251 cells using lentiviral-based shRNA (shCDO; Supplementary Fig. S4B). The addition of l-cystine to shCDO cells no longer modulated cellular respiration, whereas CSA continued to maintain its activity in this line (Fig. 2D and Supplementary Fig. S5A). To determine whether l-cystine–induced changes in respiration were attributed to cellular uptake through the system x_c_^−^ transporter, we pretreated cells with the system x_c_^−^ inhibitor sulfasalazine (13), which completely abrogated the influence of l-cystine on oxygen consumption (Fig. 2E). Furthermore, the observed CSA-induced changes in OCR were not observed with the addition of hypotaurine, the downstream metabolite of CSA (Supplementary Fig. S3C). Collectively, these findings support the concept that cysteine catabolism, through the CDO regulatory axis, attenuates cellular respiration in glioblastoma cells.

We went on to determine whether CSA-induced attenuation of cellular respiration translated to affecting other aspects of mitochondrial function. Pyruvate entry into the mitochondria allows for the generation of ATP through the electron transport chain, which results in an efflux of H^+^, leading to decreased mitochondrial membrane potential (22, 23). As demonstrated in Fig. 2F, the addition of pyruvate led to an expected increase in ATP generation, which was significantly inhibited by both CSA and l-cystine. To study mitochondrial potential, we used the JC-1 assay (24), which is a fluorescent dye that changes from red to green as the mitochondrial membrane potential decreases, signifying an increase in oxidative phosphorylation. Initial studies used the metabolic modulator dichloroacetate to serve as proof-of-concept. Dichloroacetate has been previously shown to inhibit PDH kinase (PDK), which is an endogenous inhibitor of PDH. By inhibiting PDK, dichloroacetate in turn activates PDH, allowing for increased glycolytic flux into the mitochondria and decreased mitochondrial membrane potential (23). The addition of dichloroacetate resulted in an expected increase in oxygen consumption (Supplementary Fig. S5B) and decrease in red:green ratio (Fig. 2G and Supplementary Fig. S5C), corresponding to a decrease in mitochondrial membrane potential and increase in oxidative phosphorylation, which is consistent with its previously described mechanism of action (23). Next, we evaluated the role of CSA in modulating pyruvate-induced changes in mitochondrial potential. Similar to the metabolism findings, pyruvate addition led to an expected increase in oxidative phosphorylation, resulting in a decrease in mitochondrial membrane potential. The addition of CSA in these conditions increased the red: green ratio, consistent with an increase in membrane potential and decreased oxidative phosphorylation (Fig. 2G and Supplementary Fig. S5C).

### CSA inhibits PDH activity

**Figure 3:**
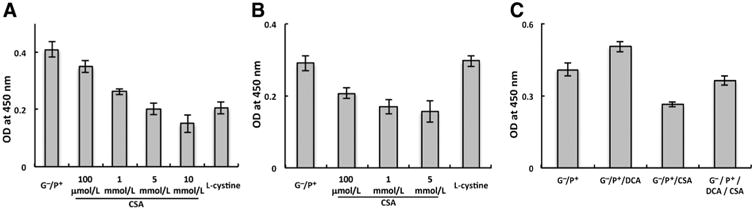
CSA inhibits PDH activity in glioblastoma cells. A, CSA and l-cystine lead to a dose-dependent inhibition of PDH activity in U251 (*P* < 0.007). B, CSA (*P* < 0.005) but not l-cystine (*P* = 0.67) inhibits PDH activity in the non-CDO–expressing cell line T98G. C, dichloroacetate (DCA; 1 mmol/L) increases PDH activity in U251 (*P* = 0.003), which is abrogated with the pretreatment of CSA (1 mmol/L; *P* = 0.117). Results are representative of at least three independent experiments.

We went on to determine the mechanism by which CSA inhibits cellular respiration in our described models. Oxidative phosphorylation involves pyruvate entry into the mitochondria, which is metabolized through the citric acid cycle. This results in the generation of NADH and FADH2, which are oxidized by the electron transport chain, resulting in a proton gradient. Oxygen serves as the terminal electron acceptor and reduced to H_2_O by cytochrome C oxidase. The resulting proton gradient generated by the electron transport chain drives the formation of ATP by the enzyme ATP synthase. As an initial investigation, we isolated mitochondria in U251 cells to determine whether the influence of CSA on oxygen consumption involved inhibiting one of these intramitochondrial steps of oxidative phosphorylation. As demonstrated in Supplementary Fig. S5D, the addition of pyruvate, but not glucose, had an expected increase in oxygen consumption in isolated mitochondria. As opposed to whole cells, the addition of CSA did not influence the OCR in isolated mitochondria. This suggests that CSA modulates OCR by inhibiting pyruvate entry into the mitochondria. Therefore, we evaluated the potential of CSA to inhibit PDH activity, which represents a gate-keeping enzyme regulating glycolytic flux into the mitochondria. As demonstrated in Fig. 3A, the addition of CSA to U251 resulted in a dose-dependent inhibition of PDH, with a maximal inhibition of approximately 64% ± 4%. Similar to the above findings involving cellular respiration, the addition of the upstream metabolite l-cystine resulted in a similar inhibition of PDH activity in the CDO1-expressing cell line U251. The inhibition of PDH activity by CSA was also observed in T98G cells; however, l-cystine did not inhibit PDH activity in this non-CDO1–expressing cell line (Fig. 3B), further supporting the role this metabolic pathway plays in the observed inhibition of PDH activity. As proof-of-concept, we did similar studies using dichloroacetate. As expected, the addition of dichloroacetate in these conditions resulted in increased PDH activity of approximately 23% ± 2% (Fig. 3C). Interestingly, the observed increase in PDH activity was abrogated when dichloroacetate was combined with CSA, further supporting the role this metabolite plays in inhibiting oxidative phosphorylation through the modulation of PDH activity.

### Inhibiting CDO attenuates glioblastoma growth *in vivo*

**Figure 4:**
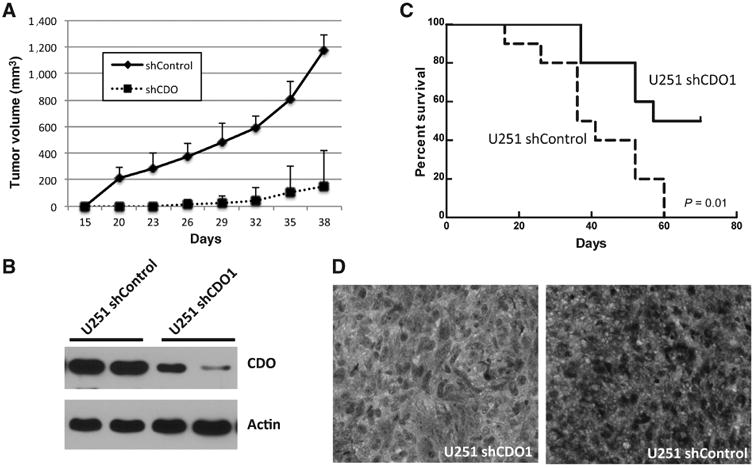
CDO modulates glioblastoma growth *in vivo*. A and B, U251 cells with stable knockdown of CDO1 using shRNA (shCDO; *n* = 5) or vector control (shControl; *n* = 5) were injected subcutaneously in a mouse flank model. Tumor growth curves were obtained at stated time points using perpendicular diameter measurements of each tumor with digital calipers and volumes calculated using the formula **(**L × W × W)/2. B, CDO1 expression determined by Western blot analysis of the indicated tumors grown in the mouse flank model. C, intracanalicular tumors were generated by implanting shCDO (*n* = 10) or shControl (*n* = 10) cells. Mice were euthanized once neurologic symptoms became apparent and survival plotted. D, CDO1 expression determined by IHC of the indicated tumors grown in an intracanalicular model.

Finally, we determined whether the CDO axis contributes toward glioblastoma growth. As an initial investigation, we characterized U251 shCDO cells *in vitro*. Cellular proliferation, colony forming capacity, and baseline levels of apoptosis and redox stress were unchanged in these lines when compared with the parental and vector control lines (Supplementary Fig. S6A–S6D). Next, we evaluated *in vivo* growth rate using a subcutaneous mouse xenograft model. As demonstrated in Fig. 4A, U251 shCDO cells had a significantly decreased growth when compared with shControl (*P* = 0.004), with Western blot analysis performed on excised tumors demonstrating a 67% and 91% decrease in CDO1 expression (Fig. 4B). We then extended these studies to evaluate survival using an intracanalicular model. U251 shCDO cells led to increased survival when compared with vector control (*P* = 0.01), with IHC performed on tumor sections confirming decreased CDO1 expression in these tumors (Fig. 4C and D).

## Discussion

A renewed interest in the study of tumor metabolism has led to several seminal discoveries in *Cancer Research*. Of these, perhaps one of the most notable has been the identification of mutations in the metabolic enzyme IDH1 in low-grade glioma and secondary glioblastoma (5). This led to a series of contributions underscoring the elegant complexity of tumorigenesis, linking a genetic mutation with the formation of a neomorphic enzyme and novel oncometabolite, 2-HG (3), which in turn, orchestrates specific epigenetic programs defining a glioma subtype (4). However, metabolic changes contributing toward the aggressive phenotype typical of high-grade glioma are only beginningtobe recognized. The majority of our understanding of glioblastoma metabolism has been provided by magnetic resonance spectroscopy. Malignant glioma has been shown to demonstrate a high resonance in the choline spectral peak and a low NAA (N-acetylaspartate) or creatine resonance correlating with high choline/NAA or choline/creatine ratios for areas of active tumors (25). In addition to choline, relative concentrations of alanine, glycine, and phosphatidylethanolamine have also been implicated in glioblastoma metabolism (26). Global metabolomic profiling has provided further insight into metabolic programs driving the aggressive phenotype of glioblastoma, along with unique metabolic subtypes with biologic and clinical application (9).

In this report, we describe the discovery of cysteine catabolism through CDO1 expression and biosynthesis of the metabolite CSA as a novel metabolic pathway in glioblastoma. Specifically, we identified grade-specific changes in CSA levels, with significant intratumoral concordance of CSA with expression of its synthetic enzyme, CDO1. Furthermore, quantitative increases in CSA between grade 2 and grade 4 glioma were the highest of all metabolites globally profiled. Interestingly, these studies identified the metabolite 2-HG as the highest fold decrease in grade 4 glioma when compared with grade 2 glioma, which is supported by recent studies (3) and reinforces the concept that unique metabolic programs underlie glioma grade.

The relevance of cysteine metabolism in cancer has gained considerable interest in recent years. The generation of GSH from cysteine, and its associated role in modulating redox status in tumors, has been well documented. Chung and colleagues (13) demonstrated the importance of this axis in glioma models, with further investigations identifying a particular relevance of this pathway in hypoxic conditions (14). They went on to show cystine uptake was via the system x_c_^−^ transporter, which mediates the exchange of extracellular cystine and intracellular glutamate, and blocking this exchange attenuated tumor growth *in vivo*. Interestingly, the glutamate exchanged during this process seems to also provide gliomas with a survival advantage, causing excitotoxic death of neurons in the vicinity of the tumor (27). Furthermore, the system x_c_^−^ transporter itself is currently being investigated as a novel tumor–specific imaging target, demonstrating the capacity to localize to areas of oxidative stress (28). Our findings, which identify utilization of this alternate, parallel pathway of cysteine catabolism in aggressive brain tumors, provide an additional layer of insight into the contributory role this emerging metabolic pathway has on gliomagenesis. In addition, these findings support the potential for both system x_c_^−^ and CDO to serve as therapeutic targets in glioblastoma.

The potential of CSA to serve as neurotransmitter has been studied (29), although its role in tumorigenesis is largely undefined. We performed a series of investigations demonstrating the potential of CSA to attenuate oxidative phosphorylation, as measured by OCR, mitochondrial membrane potential, and ATP production, through inhibition of PDH activity. Interestingly, attenuated oxidative phosphorylation is emerging as a common metabolic phenotype involved in carcinogenesis. Anso and colleagues (30) identified the capacity of c-MYC to attenuate both baseline OCR and spare respiratory capacity in osteogenic sarcoma models and Sandulache and colleagues (31) identified a diminished spare respiratory capacityinp53 mutated head and neck cancer models. Specific to glioblastoma, Vlashi and colleagues (32) identified a similar phenotype in tumor stem cells and progenitor cells when compared with their differentiated lineages. Despite these findings, the selective growth advantage offered by regulating maximal cellular respiration remains unclear. In our models, targeting the CDO/CSA pathway only inhibited glioblastoma growth *in vivo*. Therefore, we hypothesize that activation of this metabolic pathway may allow for cellular adaptation to the glioma microenvironment. With the established antioxidant role of GSH generated from cysteine in modulating redox stress, it is tempting to speculate that this parallel pathway offers these rapidly dividing tumors another layer of protection from endogenous redox damage though decreased production of free radicals generated during oxidative phosphorylation. In addition, inhibiting pyruvate entry into the mitochondria via PDH inhibition may allow for shunting of glycolytic carbon away from oxidative phosphorylation, potentially contributing to the Warburg effect and the anabolic phenotype identified in this malignancy (9). Although the effects of CSA on mitochondrial function was identified in the present study, continued investigations designed to determine how cooption of this metabolic pathway in glioblastoma may influence other aspects of gliomagenesis, including epigenetic and transcriptional programs, are still required.

Interestingly, two recent reports identified methylation and silencing of CDO1 as a common event in tumorigenesis, suggesting its role as a tumor suppressor gene (33, 34). Specifically, Dietrich and colleagues (34) identified CDO1 promoter methylation in breast cancer and demonstrated its capacity to serve as a predictive factor for distant metastases. Using a microarray-based approach, Brait and colleagues (33) identified CDO1 expression to be modulated in colorectal cancer cell lines by promoter methylation and following treatment with the DNA demethylating agent 5-aza-2′-deoxycytidine. A high frequency of CDO1 promoter methylation in colon cancer was then identified when compared with normal colon, and findings were extended to several other malignancies, including breast, esophagus, lung, bladder, and gastric cancer. They went on to show that forced expression of CDO1 resulted in decreased growth *in vitro* and *in vivo*. In contrast with these recent studies suggesting attenuating this pathway contributed toward tumorigenesis, our findings demonstrate activation of the CDO1/CSA axis is a common metabolic phenotype glioblastoma. Cysteine catabolism through the CDO1/CSA pathway is also well documented in normal brain, which has largely focused on its role in taurine synthesis (10). Interestingly, regional localization of CDO in rat brain identified this enzyme to solely localize in neurons, supporting its potential to serve as a neurotransmitter (35). We confirmed the relative increase of CDO1 expression in glioblastoma when compared with normal brain using the TCGA database (36), identifying approximately a quartile of glioblastoma to exhibit a significant increaseinCDO1 expression when compared withnormal brain (Supplementary Fig. S7A), which is consistent with our data when comparing these tumors to low-grade glioma. In addition, low levels of CDO1 expression was confirmed in normal brain sections using IHC (Supplementary Fig. S7B). Therefore, in the context of these recent publications identifying the contributory role of CDO1 as a tumor suppressor gene, our findings suggest co-option of this pathway is likely cell type–dependent and catabolism through this axis may be uniquely relevant in glioblastoma biology and its associated tumor microenvironment.

In summary, we identified a novel, targetable metabolic pathway involving cysteine catabolism in glioblastoma. These findings serve as a framework for future investigations designed to determine its clinical application in both the imaging and treatment of brain tumors and the contributory role this metabolic pathway has in tumorigenesis.

## Supplementary Material

Supplementary Figure 1

Supplementary Figure 2

Supplementary Figure 3

Supplementary Figure 4

Supplementary Figure 5

Supplementary Figure 6

Supplementary Figure 7

Supplementary Figure Legend

Supplementary Table 1
