# Supplementary figures and images for: Cysteine Catabolism: A Novel Metabolic Pathway Contributing to Glioblastoma Growth
Source: Cancer Res. Author manuscript; Available in PMC 2017 Dec 12. (PMC5726254; doi:10.1158/0008-5472.CAN-13-1423)

**A**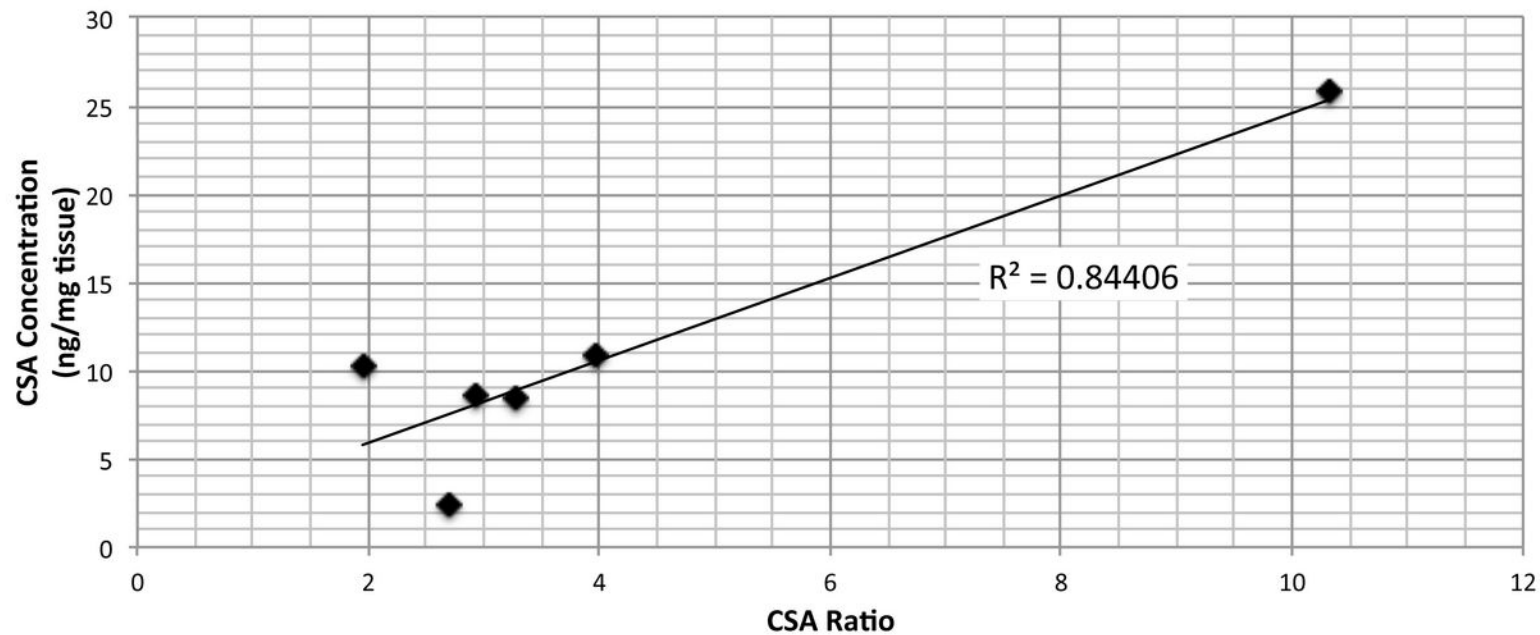**B**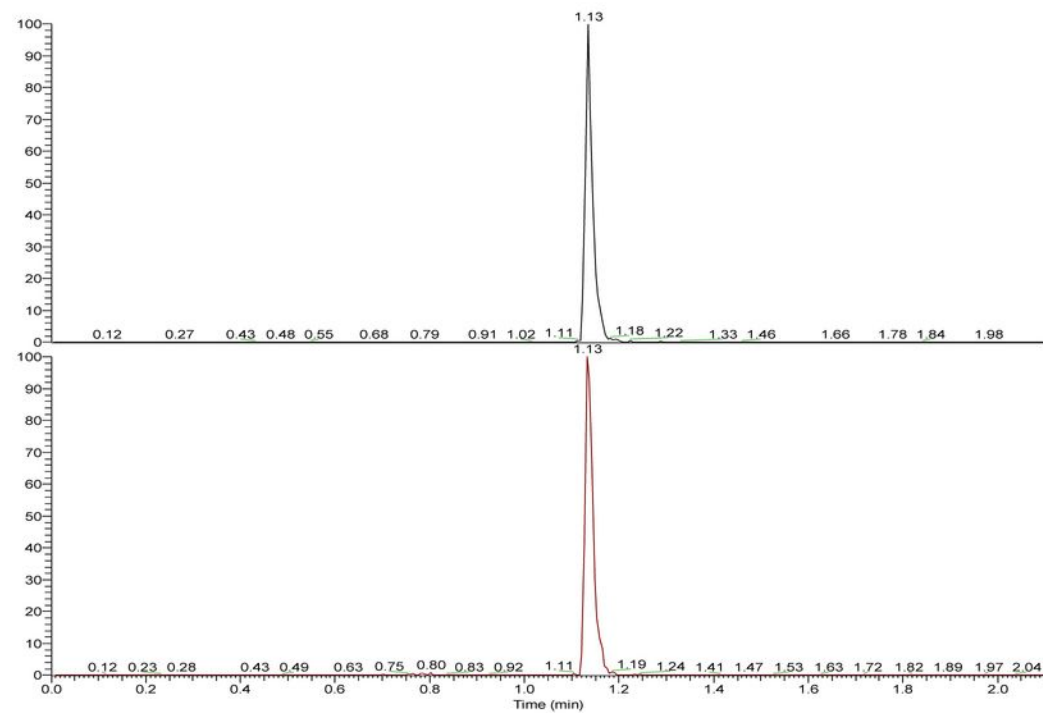**Supplementary Figure 1**

Supplement: Supplementary Figure 1 [file NIHMS717877-supplement-Supplementary_Figure_1.pdf]

**A**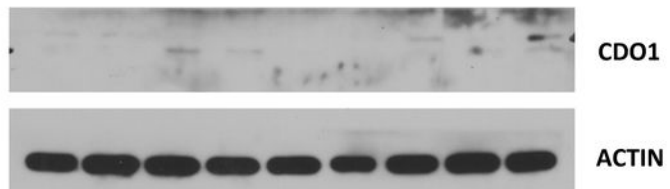**B**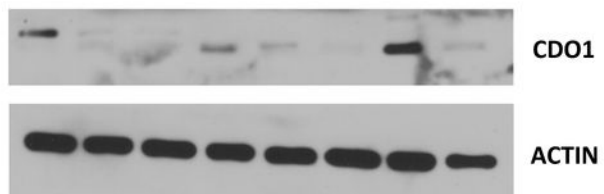**C**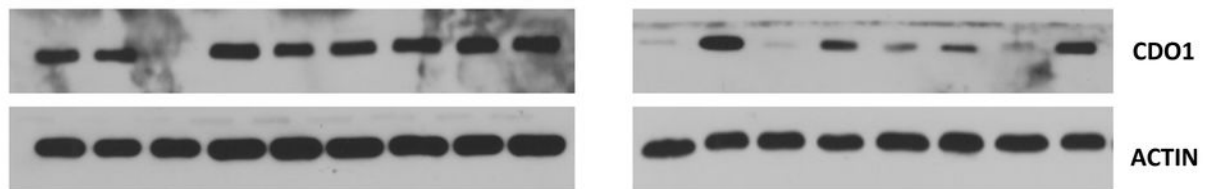

Supplement: Supplementary Figure 2 [file NIHMS717877-supplement-Supplementary_Figure_2.pdf]

**A**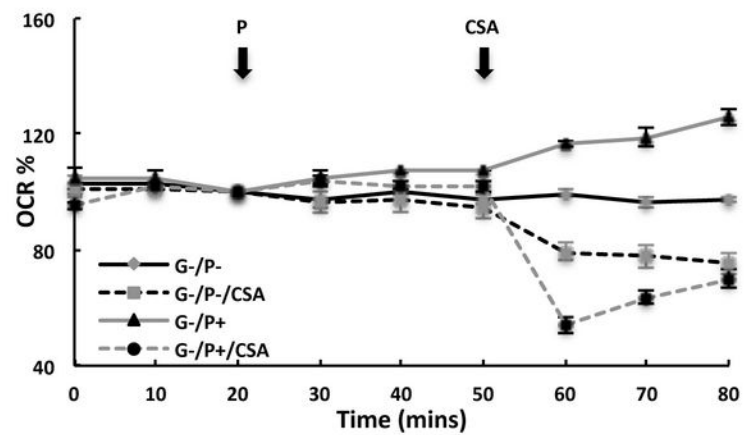**B**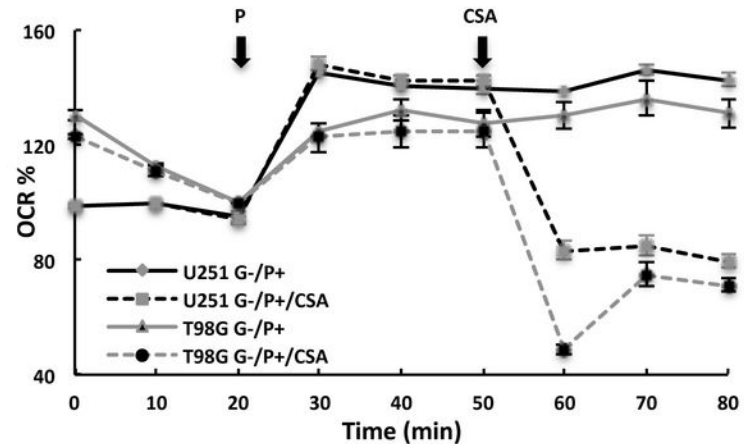**C**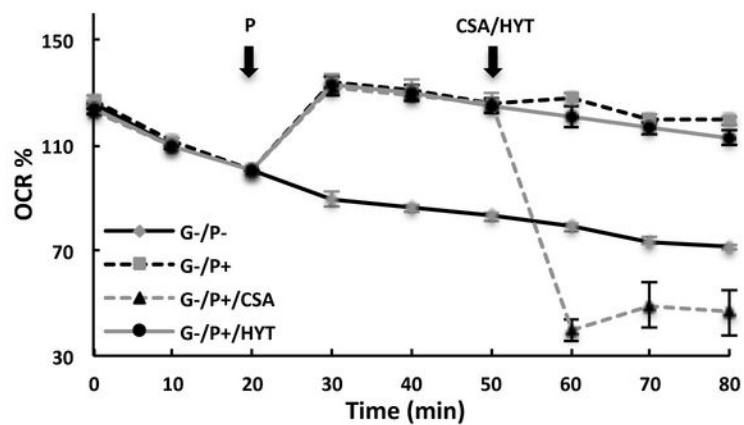

Supplement: Supplementary Figure 3 [file NIHMS717877-supplement-Supplementary_Figure_3.pdf]

**A**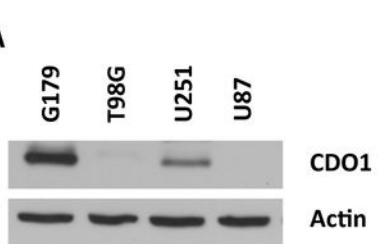**B**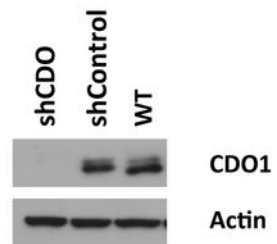

Supplement: Supplementary Figure 4 [file NIHMS717877-supplement-Supplementary_Figure_4.pdf]

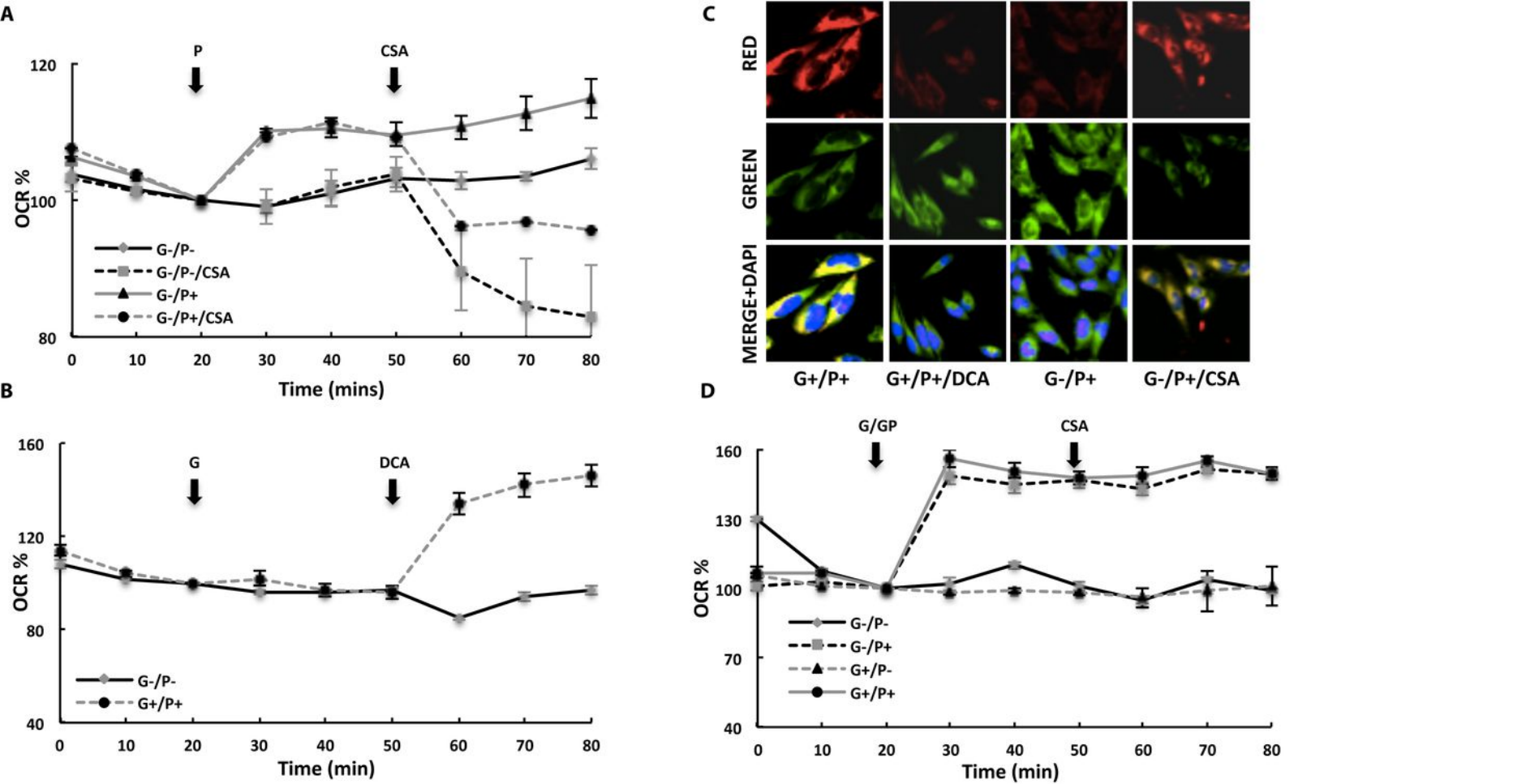

Supplementary Figure 5

Supplement: Supplementary Figure 5 [file NIHMS717877-supplement-Supplementary_Figure_5.pdf]

**A**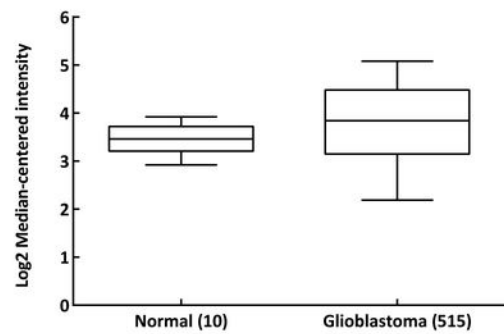**B**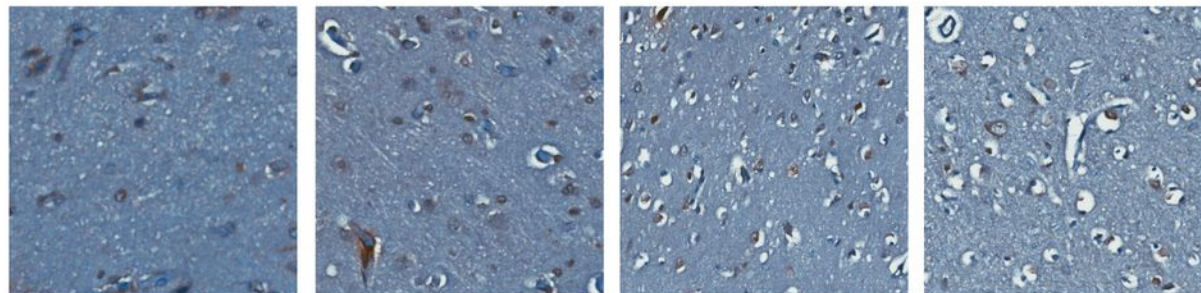

Supplement: Supplementary Figure 7 [file NIHMS717877-supplement-Supplementary_Figure_7.pdf]
