# Supplementary material for: Cysteine Catabolism: A Novel Metabolic Pathway Contributing to Glioblastoma Growth
Source: Cancer Res. Author manuscript; Available in PMC 2017 Dec 12. (PMC5726254; doi:10.1158/0008-5472.CAN-13-1423)
Supplement: Supplementary Figure 6 [file NIHMS717877-supplement-Supplementary_Figure_6.pdf]

A

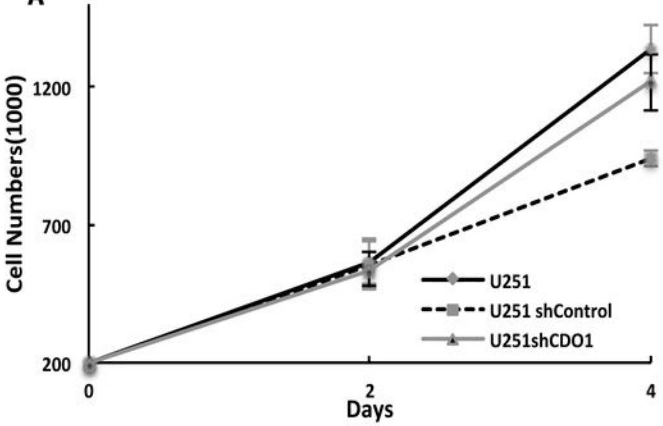

B

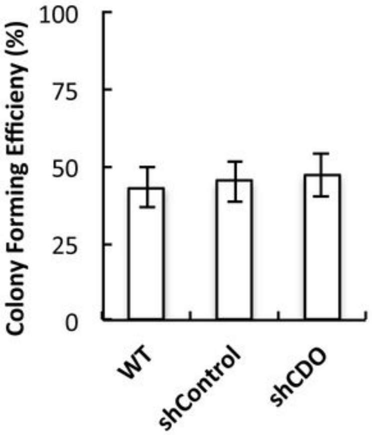

C

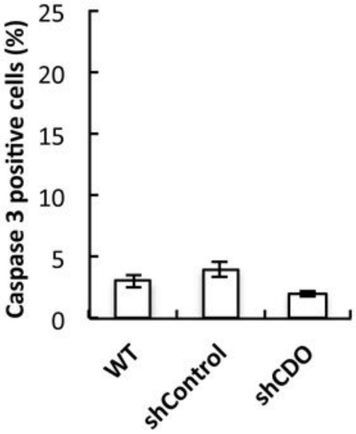

D

		Median FL 1-H
WT	baseline	445
	H202	2072
	unstained	21.4
shControl	baseline	281
	H202	1446
	unstained	9.31
shCDO1	baseline	396
	H202	1625
	unstained	11.5

Supplementary Figure 6
