# Supplementary material for: Cysteine Catabolism: A Novel Metabolic Pathway Contributing to Glioblastoma Growth
Source: Cancer Res. Author manuscript; Available in PMC 2017 Dec 12. (PMC5726254; doi:10.1158/0008-5472.CAN-13-1423)
Supplement: Supplementary Figure Legend [file NIHMS717877-supplement-Supplementary_Figure_Legend.pdf]

SUPPLEMENTARY FIGURE LEGENDS

Figure S1. CSA concentrations in GBM. (A) CSA was quantified in GBM using targeted UHPLC-MS/MS and levels were correlated with relative CSA levels obtained from global metabolomic studies. **(B)** Representative chromatograph of a tissue sample (10.3 ng CSA/mg tissue).

Figure S2. CDO1 expression in glioma. Western blot was performed on **A)** Grade II, **B)** Grade III, and **C)** Grade IV glioma tissue lysates and immunoblotted for CDO1 and actin expression.

Figure S3. CSA modulates mitochondrial function in GBM cells. A) CSA (1 mM) attenuates OCR in G179 cells in described conditions. G-/P- vs. G-/P-/CSA: p=0.0355; G-P+ vs. G-/P+/CSA: p=0.0057. **B)** CSA (1 mM) attenuates OCR in U251 (p=0.007) and T98G (p<0.001) cells in described conditions. **C)** Hypotaurine does not influence OCR in U251 cells. Results are representative of at least three independent experiments. G: glucose; P: pyruvate; CSA: cysteine sulfinic acid, HYT: hypotaurine.

Figure S4. (A) CDO1 expression in described GBM cell lines. **(B)** CDO expression in U251 wild-type (WT), cells with stable knock-down of CDO using shRNA (shCDO) and vector control (shControl). Results are representative of at least three independent experiments.

Figure S5. (A) CSA attenuates oxygen consumption rate (OCR) in U251 stable CDO knockdown cells. G-/P- vs. G-/P-/CSA : p=0.006; G-/P+ vs. G-/P+/CSA: p=0.006. **(B)** DCA (1 mM) increases OCR in U251 cells (p=0.007). **(C)** Immunofluorescent analysis of mitochondrial potential using the JC-1 assay. Fluorescent changes from red to green signify decreased mitochondrial potential and an increase in oxidative phosphorylation. **(D)** CSA does not influence OCR in isolated mitochondria. Results are representative of at least three independent experiments. G: glucose; P: pyruvate; CSA: cysteine sulfinic acid; DCA: dichloroacetate.

Figure S6. (A) Cell proliferation, **(B)** colony forming capacity, **(C)** baseline apoptosis, and **(D)** baseline and H202 induced redox stress was determine in U251 shCDO cells, compared to WT and/or shControl. The median fluorescence at baseline and following exposure to H_2O_2, which served as a positive control, is provided. Results are representative of at least three independent experiments.

Figure S7. (A) CDO1 expression in normal brain and glioblastoma evaluated on the TCGA database. **(B)** CDO1 expression in normal brain sections using IHC.

SUPPLEMENTARY TABLE LEGEND

Table 1. Top ten metabolites with highest fold increases (red) and decreases (green) in GBM when compared to Grade II glioma.
