# Supplementary material for: Cysteine Catabolism: A Novel Metabolic Pathway Contributing to Glioblastoma Growth
Source: Cancer Res. Author manuscript; Available in PMC 2017 Dec 12. (PMC5726254; doi:10.1158/0008-5472.CAN-13-1423)
Supplement: Supplementary Table 1 [file NIHMS717877-supplement-Supplementary_Table_1.pdf]

Table 1

Top ten metabolites with highest fold increases (red) and decreases (green) in GBM when compared to Grade II glioma*			
cysteine sulfinic acid	23.29	2-hydroxyglutarate	0.02
3-aminobutyrate	9.4	arabitol	0.11
2-aminoadipate	9.39	glycerophosphoethanolamine	0.11
5,6-dihydrouracil	7.82	7-alpha hydroxycholesterol	0.14
phosphoenolpyruvate	7.25	arabinose	0.16
hypotaurine	6.63	7-beta hydroxycholesterol	0.18
ribose 5-phosphate	5.78	glycerol 3 phosphate	0.18
kynurenine	4.9	glycerophosphocholine	0.19
p-cresol sulfate	4.57	arabonate	0.22
gamma-tocopherol	4.22	homocarnosine	0.23
* *P and Q values for all listed metabolites <0.001*			
